# Long-Term Survival and Quality of Life After Fenestrated Endovascular Repair for Complex Abdominal Aortic Aneurysms

**DOI:** 10.1177/15385744231154123

**Published:** 2023-02-20

**Authors:** Wietske M. A. Schreuder, Martijn L. Dijkstra, Gerdine C. I. von Meijenfeldt, Ignace F. J. Tielliu, Clark J. Zeebregts, Ben R. Saleem, Maarten J. van der Laan

**Affiliations:** Department of Surgery (Division of Vascular Surgery), 10173 University Medical Center Groningen, University of Groningen, Groningen, The Netherlands

**Keywords:** Aortic aneurysm, abdominal, endovascular procedures, blood vessel prosthesis, postoperative complications, mortality, quality of life, stents

## Abstract

**Objectives:**

Fenestrated endovascular repair (FEVAR) has become a widely used treatment option for complex abdominal aortic aneurysms (AAA) but long-term survival and quality of life (QoL) outcomes are scarce. This single center cohort study aims to evaluate both long-term survival and QoL after FEVAR.

**Methods:**

All juxtarenal and suprarenal AAA patients treated with FEVAR in a single-center between 2002 and 2016 were included. QoL scores, measured by the RAND 36-Item Short Form Survey (SF-36), were compared with baseline data of the SF-36 provided by RAND.

**Results:**

A total of 172 patients were included at a median follow-up of 5.9 years (IQR 3.0-8.8). Follow-up at 5 and 10 years post-FEVAR yielded survival rates of 59.9% and 18%, respectively. Younger patient age at surgery had a positive influence on 10-year survival and most patients died due to cardiovascular pathology. Emotional well-being was better in the research group as compared to baseline RAND SF-36 1.0 data (79.2 ± 12.4 vs 70.4 ± 22.0; P < 0.001). Physical functioning (50 (IQR 30–85) vs 70.6 ± 27.4; P = 0.007) and health change (51.6 ± 17.0 vs 59.1 ± 23.1; P = 0.020) were worse in the research group as compared to reference values.

**Conclusions:**

Long-term survival was 60% at 5-years follow-up, which is lower than reported in recent literature. An adjusted positive influence of younger age at surgery was found on long-term survival. This could have consequences for future treatment indication in complex AAA surgery but further large-scale validation is necessary.

## Introduction

Juxtarenal and suprarenal abdominal aortic aneurysms (AAA) are characterised by complex anatomy extending up to or above the renal arteries.^1,2^ This precludes the use of standard infrarenal endovascular devices as visceral vessel patency must be preserved.^1^ Over the past two decades, fenestrated, branched and other endovascular techniques have evolved from standard endovascular aneurysm repair (EVAR) to maintain visceral vessel perfusion during complex AAA treatment.^1,3^ The implementation of fenestrated endovascular aneurysm repair (FEVAR) has increased over time and the number of reported FEVAR procedures in juxtarenal AAA is approaching that of open surgical repair (OSR), which has been the leading treatment modality in complex AAA.^4,5^ It has been demonstrated that perioperative mortality was lower after FEVAR as compared to OSR.^6^ Meta-analyses of long-term mortality rates after FEVAR are scarce because of inconsistent short- and long-term follow-up in FEVAR studies.^1,6–8^ In addition to traditional outcome parameters such as morbidity and mortality, the performance of an intervention is increasingly measured through quality of life (QoL) scores as well.^7^ QoL enables impact measurement after intervention from a patient perspective. QoL can be computed by the validated RAND 36-Item Short Form Survey (SF-36), which measures self-perceived well-being over eight health concepts.^9^ The score of a health concept increases in accordance with better patient-perceived well-being.^9^

In the Netherlands, AAA patients experienced increased anxiety, worse physical functioning and worse general health after EVAR in comparison to the general Dutch population above 65 years of age.^10^ QoL scores after FEVAR are currently only available in grouped analyses for F-BEVAR.^11–13^

The aim of this study was to evaluate baseline data of long-term survival and QoL after FEVAR. By doing so, reference data for follow-up research will be provided which could aid patient tailored shared decision making. Long-term survival and QoL data have the potential to optimize patient expectations in complex AAA care and could have clinical impact on treatment implication.

## Material and Methods

### Study Design

A single-center cohort study was conducted based on prospectively collected data on complex AAA patients treated with FEVAR in a tertiary care facility in the Netherlands.^14^

Patients who underwent FEVAR between 2002 and 2016 because of complex AAA configuration were extracted from the Electronic Health Records. This specific cohort was used to pursue a minimal follow-up of 5 years. Complex AAA anatomy was defined as an infrarenal neck length of <10 mm or an AAA extending proximal to the renal arteries, but not involving the thoracic aorta.

Thoracic and thoracoabdominal aortic aneurysms were thus excluded from the analysis. Branched procedures were also excluded, as the main goal of this study was to evaluate the performance of FEVAR. The Institutional Review Board provided a waiver for this non-WMO study in accordance with the Dutch law on patient-based medical research (WMO) obligations (reference no. M21.284105). Written informed consent was obtained of all survey respondents. Patient data was processed and electronically stored in agreement with the Declaration of Helsinki’s ethical principles for medical research involving human subjects (2013). Data was stored and analyzed anonymously. The Dutch national population registration was consulted for survival status in the research group and the Dutch Central Office of Statistics (CBS) was consulted for cause of death.

### Study Outcomes

A total of 172 out of 208 patients met the inclusion criteria. Primary outcomes were long-term patient survival at a minimal follow-up of 5 years and QoL. Secondary outcome measures were short-term survival at follow-up periods of 30 days, 1 year and 3 years to provide context for long-term survival. The possible effects of preoperative patient characteristics on survival, cause of death and supplemental survey questions were secondarily studied as well.

Quality of life was measured by means of the RAND 36-Item Short Form Survey version 1.0 (RAND SF-36 1.0).^15^ Six questions were added by the research group in coordination with a health expert in the field of elderly care to evaluate care dependency and living status [Appendix A]. Care dependency could be of great added value to QoL to provide a comprehensive and patient tailored indication of treatment outcome. Data on long-term QoL in conjunction with care dependency and living status after FEVAR could enhance patient expectations of treatment outcome and therefore shared decision making.

The survey participants were asked to record their past care dependency and living status retrospectively (preoperative and 6-weeks postoperative) and during survey completion. Two more questions were added to evaluate whether patients related changes in their health status to the AAA procedure and whether they would have undergone the procedure again at that point in time when looking back to their postoperative course.

### Statistical Analysis

Categorical outcomes were expressed as frequencies and compared by the Fisher’s exact test. Continuous variables were tested for normality through the Kolmogorov-Smirnov test and by graphical analyses of summary statistics (histogram plots). Normally distributed variables were expressed as means ± standard deviation (SD) and compared though the student’s t test. Skewed variables were presented as medians with the corresponding interquartile range (IQR, Q1-Q3) and compared by the Mann Whitney U test. Odds Ratios (OR) were computed for every preoperative patient characteristic by means of univariable binary logistic regression analysis. Survival status was determined at one moment in time at a minimum follow-up of 5 years from intervention, so follow-up time variated between patients. Survival was presented through Kaplan-Meier (Log Rank test) and follow-up and survival time by descriptive statistics. A multivariable logistic regression analysis was also performed in order to present adjusted effects of preoperative patient characteristics on long-term survival per age group at operation per 5 years.

QoL was measured at the same moment in time as survival status, so again follow-up time between FEVAR and completion of the QoL survey varied from patient to patient. Normality was examined graphically (histogram) and through summary statistics over the eight health concepts and the category ‘health change’ constituted by the RAND SF-36 1.0.^16^ Baseline SF-36 1.0 values provided by RAND derived from the Medical Outcomes Study (MOS, 2471 participants) were used as reference values.^16^ Normally distributed health concept scores were compared through the one sample t-test whereas skewed distributions were compared with the reference values by the Wilcoxon Signed-Rank Test. Change in living status and care dependency of survey participants over time was analysed by the Friedman test.

All analyses were performed in IBM SPSS 23 and all survival analyses were also performed in Stata by a second researcher.

## Results

A total of 172 complex AAA patients met the inclusion criteria. The majority of this research group had juxtarenal AAA configuration (163, 94.8%), the remainder was classified as suprarenal AAA 9 (5.2%). Median aneurysm size was 61.0 mm (IQR 57.0-67.9). The Cook Zenith Fenestrated endograft was implemented in most patients (159, 92.4%), the remainder was treated with the Fenestrated Anaconda^TM^ endograft (13, 7.6%). There were 6 (3.5%) reintervention procedures after previous EVAR. Standard antiplatelet therapy consisted of 6 months of acetylsalicylic acid and life-long clopidogrel treatment.

### Preoperative Patient Characteristics

Of the included 172 patients, 44 (25.6%) patients were alive at a median follow-up of 5.9 years (IQR 3.0-8.8). Patient age at operation appeared to be significantly younger when analyzing preoperative characteristics for patients that were still alive compared to those who had died by the end of follow-up (P < 0.001) [Table 1]. Preoperative plasma creatinine levels (P = 0.002) and American Society of Anesthesiologists (ASA) physical status classification (P = 0.002) were higher in the deceased patient group. Patients with a preoperative ASA classification score of 3 were 3.3 (95% CI 1.6-7.2; P = 0.002) times more likely to die than patients with an ASA classification of 2.

**Table 1:** Preoperative patient characteristics of the total research group at a median follow-up time of 5.9 (IQR 3.0-8.8) years.

Preoperative Patient Characteristics	Total Number of Patients 172	Alive at Follow-Up 44 (25.6%)	Deceased during Follow-Up 128 (74.4%)	Or (95% CI)	P-Value
**Age at surgery** (years)	73.2 ± 7.2	69.2 ± 8.0	74.6 ± 6.3	1.12 (1.06-1.18)	<0.001
Male Sex	147 (85.5%)	38 (86.4%)	109 (85.2%)	.91 (.34-2.44)	0.845
**BMI** (kg/m^2^)	27.6 ± 4.0	28.0 ± 3.8	27.4 ± 4.2	.96 (.86-1.08)	0.511
**Preoperative smoker** ^a^					
No	57 (33.1%)	15 (34.1%)	42 (32.8%)	Referent	
Previous	54 (31.4%)	15 (34.1%)	39 (30.5%)	.93 (.40-2.15)	0.862
Yes	55 (32%)	13 (29.5%)	42 (32.8%)	1.15 (.49-2.72)	0.743
**Hypertension** ^b^					
0	39 (22.7%)	11 (25%)	28 (21.9%)	Referent	
1	60 (34.9%)	16 (36.4%)	44 (34.4%)	1.08 (.44-2.66)	0.867
2	51 (29.7%)	9 (20.5%)	42 (32.8%)	1.83 (.67-5.0)	0.236
3	22 (12.8%)	8 (18.2%)	14 (10.9%)	.69 (.23-2.09)	0.510
**Presence of diabetes mellitus**	23 (13.4%)	4 (9.1%)	19 (14.8%)	1.74 (.56-5.44)	0.338
**Cardiac status** ^c^					
0	69 (40.1%)	21 (47.7%)	48 (37.5%)	Referent	
1	43 (25%)	13 (29.5%)	30 (23.4%)	1.01 (.44-2.31)	0.982
2	41 (23.8%)	8 (18.2%)	33 (25.8%)	1.81 (.71-4.56)	0.212
3	19 (11%)	2 (4.5%)	17 (13.3%)	3.72 (.79-17.56)	0.097
**Pulmonary status** ^d^					
0	109 (63.4%)	33 (75%)	76 (59.4%)	Referent	
1	24 (14.0%)	5 (11.4%)	19 (14.8%)	1.65 (.57-4.79)	0.357
2	28 (16.3%)	4 (9.1%)	24 (18.8%)	2.61 (.84-8.10)	0.098
3	11 (6.4%)	2 (4.5%)	9 (7%)	1.95 (.40-9.54)	0.408
**COPD**					
No	111 (64.5%)	32 (72.7%)	79 (61.7%)	Referent	
Mild	18 (10.5%)	5 (11.4%)	13 (10.2%)	1.05 (.35-3.20)	0.927
Moderate	29 (16.9%)	5 (11.4%)	24 (18.8%)	1.94 (.68-5.54)	0.213
Severe	13 (7.6%)	1 (2.3%)	12 (9.4%)	4.86 (.61-38.94)	0.136
Very severe	1 (.6%)	1 (2.3%)	-	-	
Unknown	63 (36.6%)	10 (22.7%)	75 (58.6%)		
**Presence of cerebrovascular disease**	28 (16.3%)	6 (13.6%)	22 (17.2%)	1.31 (.50-3.49)	0.583
**Presence of peripheral artery disease**	10 (5.8%)	2 (4.5%)	8 (6.3%)	1.91 (.38-9.52)	0.429
Unknown	63 (36.6%)	10 (22.7%)	75 (58.6%)		
**Plasma creatinine >90 μmol/L**	87 (50.6%)	13 (29.5%)	74 (57.8%)	3.27 (1.57-6.83)	0.002
**ASA classification** ^a^					
2	40 (23.3%)	18 (40.9%)	22 (17.2%)	Referent	
3	122 (70.9%)	24 (54.5%)	98 (76.6%)	3.34 (1.55-7.19)	0.002
4	4 (2.3%)	1 (2.3%)	3 (2.3%)	2.46 (.24-25.67)	0.453
**Aneurysm location**					
Juxtarenal	163 (94.8%)	41 (93.2%)	122 (95.3%)	Referent	
Suprarenal	9 (5.2%)	3 (6.8%)	6 (4.7%)	.67 (.16-2.81)	0.586

Categorical data presented as No. (%), continuous data presented as mean ± SD or median (IQR).

Abbreviations: BMI, body mass index; COPD, chronic obstructive pulmonary disease and ASA classification, American Society of Anesthesiologists physical status classification.

^a^Missing values less than 5%.

^b^Hypertension as described by Chaikof et al.^24^.(0) None (diastolic pressure (DP) < 90 mmHg), (1) Controlled with 1 drug (DP <90 mmHg), (2) Controlled with 2 drugs, (3).Controlled with >2 drugs/uncontrolled.

^c^Cardiac status as described by Chaikof et al.^24^.(0) Asymptotic; normal ECG, (1) Asymptotic; remote myocardial infarction (MI (history)), occult MI (ECG) or fixed defect (dipyridamole thallium or similar scan), (2) Stable angina; reversible perfusion defect (scan); silent ischemia (1% of time); ejection fraction (EF) 25-45%; controlled ectopy/asymptomatic arrythmia; history of congestive heart failure that is now well compensated, (3) Unstable angina; symptomatic or poorly controlled ectopy/arrythmia; poorly compensated/recurrent heart failure; EF < 25%; MI ≤ 6 months.

^d^Pulmonary status as described by Chaikof et al.^24^.(0) Asymptotic; normal chest X-ray; pulmonary fraction test within 20% of predicted, (1) Asymptomatic/mild dyspnea on exertion; mild chronic parenchymal radiograph changes; pulmonary function test 65-80% of predicted, (2) Between 1 and 3, (3) Vital capacity <1.85 L; FEV1 <1.2 L/<35% of predicted; PCO2 >45 mmHg; supplementary oxygen medically necessary; pulmonary hypertension.

### Survival

**Figure 1.**
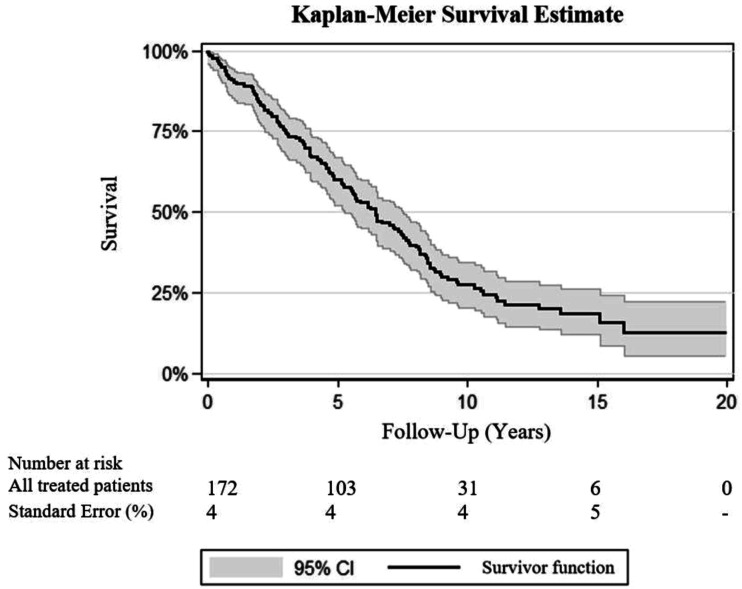
Kaplan-Meier analysis predicting long-term survival after FEVAR.

Two out of 172 (1.2%) patients died at 30 days follow-up. At follow-up periods of 1, 3 and 5 years after FEVAR, survival rates of 156 (90.7%), 129 (75%) and 103 (59.9%) were observed, respectively. Kaplan-Meier analysis demonstrated estimated survival rates of 18% at a 10-year follow-up and 3.5% at a 15-year follow-up [Figure 1].

### RAND SF-36

The QoL questionnaire was completed by 31 out of 44 (70.5%) patients who were alive at follow-up. Of the 13 (29.5%) non-responders, 3 (6.8%) patients were unable to participate due to dementia, 1 (2.3%) patient was unreachable, and the remainder did not wish to participate (9, 4.9%). Median age at follow-up of the non-responders was 82.3 (IQR 79.0-86.9) years and the majority (10, 76.9%) was male. Median age at surgery was 72.3 (IQR 66.2-75.9) years and median follow-up time was 10.8 (IQR 8.5-13.9) years in this subgroup. The majority of the 31 responders were male (28, 90.0%) and median age at follow-up was 79.1 (IQR 72.5-84.7) years. Median age at surgery was 68.3 (IQR 62.8-76.0) years and follow-up time was 9.6 (IQR 7.0-13.4) years.

Four out of nine RAND SF-36 health concepts were normally distributed and associated with mean scores of 57.6 ± 19.9 for energy/fatigue, 79.2 ± 12.4 for emotional well-being, 51.1 ± 21.6 for general health and 51.6 ± 17.0 for health change [Table 2]. The remaining health concepts had skewed distributions and came down to median scores of 50 (IQR 30-85) for physical functioning, 75 (IQR 25-100) for physical role functioning, 100 (IQR 66.7-100) for emotional role functioning, 87.5 (IQR 62.5-100) for social functioning and 87.5 (IQR 55-100) for pain.

**Table 2.** RAND SF-36 scores representing quality of life at long-term follow-up after FEVAR with reference basline data provided by RAND.

	RAND SF-36 Scores		
Health concept	Research group (31)	Medical outcomes study (2471)	P-value
Physical functioning	50 (IQR 30-85)	70.61 ± 27.42	0.007
Role functioning/physical	75 (IQR 25-100)	52.97 ± 40.78	0.906
Role functioning/emotional	100 (IQR 66.7-100)	65.78 ± 40.71	0.039
Energy/fatigue	57.6 ± 19.9	52.15 ± 22.39	0.151
Emotional well-being	79.2 ± 12.4	70.38 ± 21.97	<0.001
Social functioning	87.5 (IQR 62.5-100)	78.77 ± 25.43	0.738
Pain	87.5 (IQR 55-100)	70.77 ± 25.46	0.055
General health	51.1 ± 21.6	56.99 ± 21.11	0.142
Health change	51.6 ± 17.0	59.14 ± 23.12	0.020

Data presented as mean ± SD or median (IQR).

RAND SF-36 scores in the research group were compared to baseline data demonstrated by the RAND Corporation.^16^ It was found that emotional role-functioning (100 (IQR 66.7-100) vs 65.78 ± 40.71; P = 0.039) and emotional well-being (79.2 ± 12.4 vs 70.4 ± 22.0; P < 0.001) scored better in the research group.^16^ Physical functioning (50 (IQR 30-85) vs 70.6 ± 27.4; P = 0.007) and health change (51.6 ± 17.0 vs 59.1 ± 23.1; P = 0.020) rated worse in the research group as compared to RAND SF-36 baseline data.^16^

### Survival Subgroup Analysis

Of all baseline characteristics, only age at time of surgery was associated with risk of death during follow-up [Table 3]. Unadjusted and adjusted risks of death at a 1-, 3- and 5-year follow-up were not significantly influenced by patient age at time of surgery. Increased patient age at surgery did show an increased risk of death at 10-years follow-up.

**Table 3.** Multivariable linear regression analysis demonstrating the effect of age at surgery on survival at 5- and 10-years follow-up after FEVAR.

Risk of Death after FEVAR at Total Follow-Up (Total 172)						
Age at surgery (years)	<60	60-65	65-70	70-75	75-80	≥80
	6 (3.5%)	17 (9.9%)	28 (16.3%)	38 (22.1%)	55 (32.0%)	28 (16.3%)
Unadjusted OR	(Referent)	4.2	2.3	5.4	3.9	6.6
(95% CI)		(0.4-43.0)	(0.2-22.1)	(0.6-49.3)	(.5-34.9)	(.7-63.3)
P-value		0.227	.475	0.135	.218	.105
Adjusted^a^ OR	(Referent)	4.0	2.3	6.6	5.0	6.7
(95% CI)		(0.3-49.1)	(0.2-24.3)	(0.7-67.3)	(.5-48.7)	(.6-73.4)
P-value		0.280	0.500	0.110	.166	.121
Risk of death after FEVAR at total follow-up (total 149)						
Age at surgery (years)	<60	60-65	65-70	70-75	75-80	≥80
	6 (4.0%)	14 (9.4%)	24 (16.1%)	35 (23.5%)	51 (34.2%)	19 (12.8%)
Unadjusted OR	(Referent)	3.6	2.8	9.7	23.5	17.0
(95% CI)		(.5-27.1)	(.4-18.4)	(1.4-65.4)	(3.2-170.3)	(1.8-160.1)
P-value		0.214	0.283	0.020	.002	.013
Adjusted^a^ OR	(Referent)	4.0	1.8	19.6	64.1	23.4
(95% CI)		(.4-44.0)	(.2-15.9)	(2.1-184.4)	(5.2-784.5)	(1.7-316.4)
P-value		0.259	0.589	0.009	.001	.018

^a^Estimates adjusted for sex, hypertension, cardiac status, pulmonary status, COPD, preoperative plasma creatinine (>90 μmol/L) and ASA classification.

Patients with an age between 70 and 75 years had a 19.6 (95% CI 2.1-184.4; P = 0.009) increased risk of death at 10 years after FEVAR compared to patients <60 years old at time of surgery. In patients with ages between 75 and 80 years old and above 80 years old at time of surgery, this risk was 64.1 (95% CI 5.2-784.5; P = 0.001) and 23.4 (95% CI 1.7-316.4; P = 0.018) fold increased compared to patients <60 years old at time of surgery, respectively.

The age-category of 70-75 years old at surgery was the first age-category to show a significant difference in survival at 10-years follow-up in comparison to patients <60 years old at time of operation. Subgroup analysis by Kaplan-Meier was thus performed at a cutoff of 70 years old.

**Figure 2.**
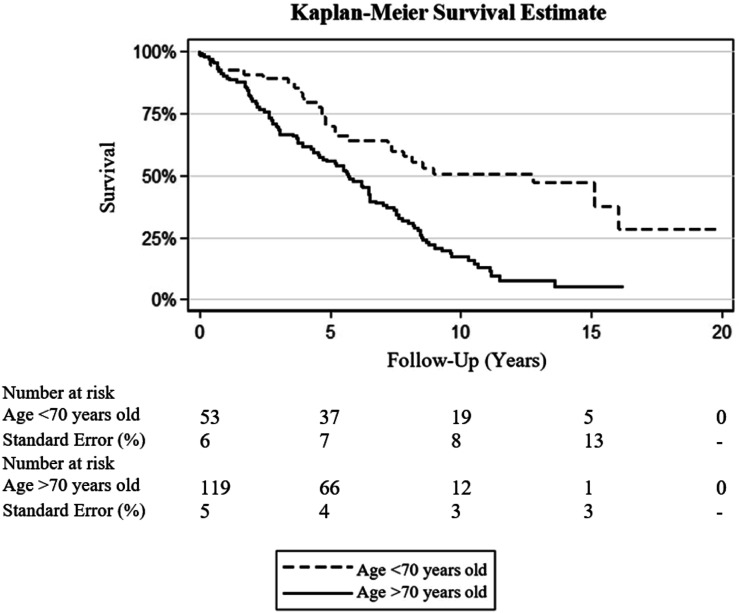
Subgroup analysis by Kaplan-Meier predicting long-term survival after FEVAR in patients with an age at operation above and below 70 years old.

Kaplan-Meier analysis demonstrated that patients aged <70 years at the time of operation (53/172, 30.8%) in comparison to >70 years old (119/172, 69.2%) were associated with better survival (P < 0.001) [Figure 2]. At a 5- and 10-year follow-up it was demonstrated that 69.8% and 35.8% of patients would still be alive for the age group <70 years old and 55.5% and 10.1% of patients >70 years at time of operation, respectively. Median survival time at follow-up was 7.6 (IQR 4.7-13.2) years for patients <70 years old vs 5.5 (IQR 2.7-8.2) for patients >70 years old.

### Cause of Death

Cause of death was mostly cardiovascular (48/128, 37.5%) and specifically cardiac (30/128, 23.4%), of which 11 (8.6%) patients died an acute cardiac death [Table 4]. Furthermore, cause of death was pulmonary in 21 (16.4%) patients and vascular in 18 (14.1%) patients. The remaining causes of death were the GI-tract/liver/pancreas (15/128, 11.7%), the brain (14/128, 10.9%), malignancies (13/128, 10.2%) and unspecified causes (17/128, 13.3%).

**Table 4.** Cause of death in the total research population at a median follow-up time of 5.9 years (IQR 3.0-8.8) after FEVAR.

Cause of Death (128)	Frequency	Subgroups	Frequency
Heart	30 (23.4%)	Acute myocardial infarction/cardiac death	11 (8.6%)
		Other heart pathology^a^	19 (14.8%)
Lungs	21 (16.4%)	Chronic obstructive lung disease^b^	11 (8.6%)
		Lung infections and unspecified lung pathology^c^	10 (7.8%)
Vascular^d^	18 (14.1%)		
GI-tract, liver, pancreas^e^	15 (11.7%)		
Brain^f^	14 (10.9%)		
Malignancy	13 (10.2%)		
Unspecified	17 (13.3%)		

Categorical data presented as No. (%).

Abbreviations: GI-tract, gastrointestinal tract.

^a^Heart decompensation, heart valve pathology, atherosclerotic heart disease, chronic ischaemic heart disease or atrial fibrillation/flutter.

^b^Chronic obstructive lung disease with or without an acute lower respiratory infection.

^c^Unspecified lung pathology, unspecified lung infection, pneumonia or the coronavirus COVID-19.

^d^Aortic atherosclerosis/dissection, aortic aneurysm, aneurysm of unspecified location, generalised/distal atherosclerosis or an (acute) vascular insult of the intestines.

^e^Gastrointestinal tract bleeding, pathology in the: oesophagus, stomach, antrum pylori, small intestines, colon, liver or pancreas, Crohn’s disease or abdominal hernia.

^f^Unspecified brain pathology, intracerebral hemorrhage/infarction, hypophysis pathology, dementia or senility.

### Care Dependency

Preoperative, 6 weeks postoperative and current living status stayed almost constant (P = 0.717) in the survey group. All survey participants lived independently of nursing facilities of whom 5 out of 31 lived alone and 26 patients lived together with others before and 6 weeks after the procedure. This ratio was 6 patients living alone and 25 patients living in the company of others at a median follow-up of 9.6 (IQR 7.0-13.4) years. Care dependency changed more over time but not significantly (P = 0.247)*.* One patient (1/31, 3.2%) responded that she related her negative health changes to the AAA procedure and that she would not choose to undergo the procedure again.

## Discussion

Of the total research group of 172 patients, 2 (1.2%) patients died within 30-days after FEVAR. Furthermore, a survival rate of 156 (90.7%) and 129 (75%) patients was observed 1 and 3 years post-FEVAR, respectively. Long-term survival was lower than demonstrated in recent reference papers, with a survival rate of 103 (59.9%) patients at 5 years follow-up.^1,17^ Survival was influenced at 10-years follow-up by patient age at surgery. Most patients died of cardiovascular causes, this could, however, be biased since acute aneurysm-related mortality is often mistaken for cardiac death in clinical practice.

All 31 survey participants lived independently in their home. The observation of better emotional role-functioning (100 (IQR 66.7-100) vs 65.78 ± 40.71; P = 0.039) and emotional well-being (79.2 ± 12.4 vs 70.4 ± 22.0; P < 0.001) in the research group as compared to the RAND SF-36 1.0 baseline data was striking considering possible postoperative complications after FEVAR.^16^ The latter consideration could on the other hand explain the decreased physical functioning (50 (IQR 30-85) vs 70.6 ± 27.4; P = 0.007) and health change (51.6 ± 17.0 vs 59.1 ± 23.1; P = 0.020) in the research group in comparinson to the reference data.^16^

Mortality rate at 30-days (1.2%) was low in comparison to reference data, which mostly report 30-day mortality rates around 2.5-3.0%.^1,6^

Survival rates at a 1-and 3-year follow-up are in accordance with most of the available literature.^13,17–19^ At a 5-year follow-up, survival rates were higher in a reference study, namely 71.0%, than in the current analysis (59.9%).^17^ This discrepancy may in part be explained by the use of the Dutch national registration to assess survival, which is not influenced by loss to follow up, as might have been the case in other studies. A recent systematic review on FEVAR in complex AAA reported an estimated 5-year survival of 78%, although this reference meta-analysis did report an inconsistency in follow-up, as did other literature studies.^1^

Deteriorating change in health and physical functioning in the present survey group as compared to the RAND SF-36 baseline data could be attributed to the FEVAR procedure. However, the majority of participants stressed that their decline in physical health was due to other comorbidities (such as COPD). Another explanation for this finding could be a mismatch in patient characteristics. The MOS was performed to set baseline QoL scores for diverse populations. It is uncertain whether the MOS reference data truly matches the current research group but the RAND Corporation did state optimalisation of the SF-36 1.0 reference data for elderly and chronically ill populations in comparison with older SF-36 reference data.^9^ The original work on SF-36 also demonstrated that relatively large differences in mental health domains were not clinically significant, yet rating differences as small as 5 demonstrated clinical relevance in physical health domains.^9^ The RAND SF-36 rating of physical and mental health domains are weakly correlated with one another.^20^

Emotional role-functioning (100 (IQR 66.7-100)) was higher in the research group in comparison to a previous study 6-8 weeks after F-BEVAR (76.9 ± 37.4).^11^ Emotional well-being (79.2 ± 12.4) and physical functioning (50 (IQR 30-85)) were comparable to outcomes in that same reference group (78.7 ± 16.8 and 51.9 ± 25.8, respectively).^11^ Health change was not reported.^11^

Previous studies have shown that age alone is a poor predictor for recovery and survival after F-BEVAR.^11,13^ This is contradicted by the present analysis in which an adjusted influence of patient age at surgery was found on 10-year survival after FEVAR. This discrepancy could be explained by a difference in length of follow-up, which was longer in the present study as compared to the reference studies. It is reasonable to assume that, even without an intervention, an old patient group will probably associate with shorter survival time after longer follow-up. The finding that an operative age at surgery below and above 70 years influenced long-term survival could be of interest in treatment indication and future shared decision making. Large scale follow-up research should be conducted to further establish the correlation of age at surgery with long-term survival.

All survey respondents lived independently of nursing facilities at a median follow-up of 9.6 (IQR 7.0-13.4) years after FEVAR. Therefore, the survey respondents did not fall under the Dutch care profiles for elderly (Zorgzwaartepakket Verpleging en Verzorging, ZZP VV).^21^ In 2020, roughly 5.1% of the Dutch elderly utilized the ZZP VV.^22,23^ It seems as if the research group had a higher independence than the average Dutch population above >65 years old but the non-response rate of 29.5% precludes further conclusions. Follow-up research in a larger sample size is needed to confirm long-term care dependency in complex AAA patients after FEVAR.

Small sample size and a non-response rate of 29.5% to the QoL survey are weak points of this study. A strength is the absence of recall bias in the QoL surveys as participants were required to rate their current QoL. Recall bias could have distorted outcomes on living situation and care dependency as this was partially queried retrospectively. The demonstration of both QoL and care dependency was considered of great potential for the establishment of future comprehensive patient centered treatment outcomes.

Selection bias (due to survivorship) is probably a profound weakness of this study. Survey respondents had relatively good mental health and independent living statuses. Yet, most patients had died at a median follow-up of approximately 6 years. A QoL sample at maximally 1- or 3-year follow-up may yield different outcomes as compared to the current analysis because the most deteriorating patient population post-FEVAR may still have been able to self-report satisfaction and physical well-being. The outcomes of this study probably represent patient-satisfaction in the vital complex AAA patient rather than the average patient population after FEVAR. This could also be the result of a relatively large amount of non-responders (29.5%) on a small survey group.

Further weakness of this study is the lack of data on aneurysm related deaths, this could be of great value in future research.

In conclusion, survival rates after FEVAR were 98.9%, 90.7% and 75% at 30 days, 1 year and 3 years follow-up, retrospectively. Long-term survival was low, namely 59.9% at a 5-year follow-up and an estimated 18% at a 10-year follow-up. This can possibly be explained by the use of the national registry for survival analysis and therefore seems to represent the real world outcomes of FEVAR. Present beliefs about long-term survival after (successful) FEVAR could thus be worse than previously reported. Particularly since a lot of clinical implications are based on 30-day outcomes, and this study found high 30-day survival rates but low 5-year survival. Follow-up research is necessary in order to investigate the long-term performance of FEVAR.

Furthermore, physical functioning and health change were worse in the research group in reference to the RAND SF-36 1.0 baseline data.^16^ QoL scores and care dependency can help to reflect the impact of treatment outcomes on patients in future research.

Lastly, a positive influence of younger patient age at time of operation on long-term survival was found. Larger sample sizes may also demonstrate a relationship between preoperative patient characteristics and long-term QoL. Future research should aim to analyze the effects of preoperative predictors of patient tailored outcome after FEVAR. This could improve future shared decision making in complex AAA patients after FEVAR.

## Appendix A

Care dependancy was rated through the following answer possibilities: (1) No cleaning nor nursing support; Help with cleaning; (3) Informal caregiver: help with activities of daily living (ADL); (4) Nurse: medical help and (5) Nurse: ADL support and medical help.

Living status was specified by the following answering options: (1) Independent (from any nursing facility), alone; (2) Independent, together (partner, family, etc.); (3) Assisted living; (4) Retirement home; (5) Nursing home for revalidation (temporary) (6) Nursing home.

## References

[1] Mohamed N Galyfos G Anastasiadou C , et al. Fenestrated Endovascular Repair for Pararenal or Juxtarenal Abdominal Aortic Aneurysms: A Systematic Review. Ann Vasc Surg 2020;63:399-408. doi:10.1016/j.avsg.2019.09.016 31629840

[2] Wanhainen A Verzini F Van Herzeele I , et al. Editor’s Choice - European Society for Vascular Surgery (ESVS) 2019 Clinical Practice Guidelines on the Management of Abdominal Aorto-Iliac Artery Aneurysms. Eur J Vasc Endovasc Surg 2019;57(1):8-93. doi:10.1016/j.ejvs.2018.09.020 30528142

[3] Sun Z Mwipatayi BP Semmens JB Lawrence-Brown MMD . Short to Midterm Outcomes of Fenestrated Endovascular Grafts in the Treatment of Abdominal Aortic Aneurysms: A Systematic Review. J Endovasc Ther an Off J Int Soc Endovasc Spec 2006;13(6):747-753. doi:10.1583/06-1919.1 17154710

[4] Končar IB Jovanović AL Dučič SM . The Role of FEVAR, CHEVAR and Open Repair in Treatment of Juxtarenal Aneurysms: A Systematic Review. J Cardiovasc Surg 2020;61(1):24-36. doi:10.23736/S0021-9509.19.11187-1 32079378

[5] Von Meijenfeldt GCI Alberga AJ Balm R , et al. Results from a Nationwide Prospective Registry on Open Surgical or Endovascular Repair of Juxtarenal Abdominal Aortic Aneurysms. J Vasc Surg 2022;75(1):81-89.e5. doi:10.1016/j.jvs.2021.06.031 34197942

[6] Patel SR Ormesher DC Griffin R Jackson RJ Lip GYH Vallabhaneni SR . Comparison of Open, Standard, and Complex Endovascular Aortic Repair Treatments for Juxtarenal/Short Neck Aneurysms: A Systematic Review and Network Meta-Analysis. Eur J Vasc Endovasc Surg 2022;63:696-706. doi:10.1016/j.ejvs.2021.12.042 35221243

[7] Doonan RJ Girsowicz E Dubois L Gill HL . A Systematic Review and Meta-Analysis of Endovascular Juxtarenal Aortic Aneurysm Repair Demonstrates Lower Perioperative Mortality Compared with Open Repair. J Vasc Surg 2019;70(6):2054-2064.e3. doi:10.1016/j.jvs.2019.04.464 31327612

[8] Ou J Chan YC Cheng SW . A Systematic Review of Fenestrated Endovascular Repair for Juxtarenal and Short-Neck Aortic Aneurysm: Evidence so Far. Ann Vasc Surg 2015;29(8):1680-1688. doi:10.1016/j.avsg.2015.06.074 26256714

[9] Steward AL Sherbourne C Hayes RD . Measuring Functioning and Well-Being: The Medical Outcome Study Approach. In: Stewart AL Ware JE , eds. Summary and Discussion of MOS Measures. Duke University Press; 1992:345-371.

[10] Geraedts ACM Mulay S Terwee CB , et al. Patient-Reported Outcomes of Yearly Imaging Surveillance in Patients Following Endovascular Aortic Aneurysm Repair. Ann Vasc Surg. Published online 2021;82:1-7. doi:10.1016/j.avsg.2021.11.003 34902477

[11] Kärkkäinen JM Sandri Gde A Tenorio ER , et al. Prospective Assessment of Health-Related Quality of Life after Endovascular Repair of Pararenal and Thoracoabdominal Aortic Aneurysms using Fenestrated-Branched Endografts. J Vasc Surg 2019;69(5):1356-1366.e6. doi:10.1016/j.jvs.2018.07.060 30714570

[12] Sen I Tenorio ER Pitcher G , et al. Effect of Obesity on Radiation Exposure, Quality of Life Scores, and Outcomes of Fenestrated-Branched Endovascular Aortic Repair of Pararenal and Thoracoabdominal Aortic Aneurysms. J Vasc Surg 2021;73(4):1156-1166.e2. doi:10.1016/j.jvs.2020.07.088 32853700

[13] Kärkkäinen JM Tenorio ER Oksala N , et al. Pre-Operative Psoas Muscle Size Combined with Radiodensity Predicts Mid-Term Survival and Quality of Life After Fenestrated-Branched Endovascular Aortic Repair. Eur J Vasc Endovasc Surg 2020;59(1):31-39. doi:10.1016/j.ejvs.2019.06.021 31718987

[14] de Niet A Donselaar EJ Holewijn S , et al. Endograft Conformability in Fenestrated Endovascular Aneurysm Repair for Complex Abdominal Aortic Aneurysms. J Endovasc Ther 2020;27(5):848-856. doi:10.1177/1526602820936185 32567964 PMC7536524

[15] RANDWare JE SC (1990); N versie: S-36: AN (1998). 36-Item Short Form Health Survey SF-36/MOS SF-36 / RAND-36. Meetinstrumenten in de Zorg. https://meetinstrumentenzorg.nl/instrumenten/36-item-short-form-health-survey/September, 2022. Accessed.

[16] RAND. 36-Item Short Form Survey (SF-36) Scoring Instructions. Scoring Rules for the RAND 36-Item Health Survey (Version 1.0). https://www.rand.org/health-care/surveys_tools/mos/36-item-short-form/scoring.html September, 2022. Accessed.

[17] Sveinsson M Sonesson B Kristmundsson T Dias N Resch T . Long-Term Outcomes After Fenestrated Endovascular Aortic Repair for Juxtarenal Aortic Aneurysms. J Vasc Surg 2022;75(4):1164-1170. doi:10.1016/j.jvs.2021.11.050 34838610

[18] Jones AD Waduud MA Walker P Stocken D Bailey MA Scott DJA . Meta-Analysis of Fenestrated Endovascular Aneurysm Repair Versus Open Surgical Repair of Juxtarenal Abdominal Aortic Aneurysms Over the Last 10 Years. BJS open 2019;3(5):572-584. doi:10.1002/bjs5.50178 31592091 PMC6773647

[19] Chinsakchai K Prapassaro T Salisatkorn W , et al. Outcomes of Open Repair, Fenestrated Stent Grafting, and Chimney Grafting in Juxtarenal Abdominal Aortic Aneurysm: Is It Time for a Randomized Trial? Ann Vasc Surg 2019;(56(November 2018):):114-123. doi:10.1016/j.avsg.2018.08.097 30476617

[20] Hays RD Sherbourne CD Mazel R . User’s Manual for the Medical Outcomes Study (MOS) Core Measures of Health-Related Quality of Life. RAND Corporation; 1995.

[21] Services M. Microdata Services Documentatie Door Het CIZ Afgegeven Indicatie Voor Wlz-Zorg. (INDICWLZTAB) Microdata Services. Centraal Bureau voor de Statistiek 2021. September.

[22] Centraal Bureau voor de Statistiek. Personen met Indicatie naar Gebruik WLZ-Zorg; Indicatie, Leveringsvorm, ZZP. https://opendata.cbs.nl/statline/#/CBS/nl/dataset/84529NED/table?ts=1646224357727 September, 2022. Accessed.

[23] Centraal Bureau voor deBevolking Statistiek. ; kerncijfers. 2021. https://opendata.cbs.nl/#/CBS/nl/dataset/37296ned/table?ts=1653906220184 September, 2022. Accessed.

[24] Chaikof EL Fillinger MF Matsumura JS , et al. Identifying and Grading Factors that Modify the Outcome of Endovascular Aortic Aneurysm Repair. J Vasc Surg 2002;35(5):1061-1066. doi:10.1067/mva.2002.123991 12021728

